# Sex-specific association between osteoporosis and cataracts: UK Biobank and Hong Kong Osteoporosis Study

**DOI:** 10.1007/s11657-026-01674-0

**Published:** 2026-02-24

**Authors:** Xiaowen Zhang, Suhas Krishnamoorthy, Kelvin K. L. Chong, Jonathan K. L. Mak, Kathryn Choon-Beng Tan, Annie Wai-Chee Kung, Ian Chi-Kei Wong, Hou-Feng Zheng, Ching-Lung Cheung

**Affiliations:** 1 Department of Pharmacology and Pharmacy, Li Ka Shing Faculty of Medicine, The University of Hong Kong, 2/F Laboratory Block, 21 Sassoon Road, Hong Kong SAR, China; 2 Department of Ophthalmology and Visual Sciences, Faculty of Medicine, The Chinese University of Hong Kong, Hong Kong SAR, China; 3 Department of Medicine, Li Ka Shing Faculty of Medicine, The University of Hong Kong, Hong Kong SAR, China; 4 Research Department of Practice and Policy, School of Pharmacy, University College London, London, UK; 5 Westlake Laboratory of Life Sciences and Biomedicine, 18 Shilongshan Road, Xihu District, Hangzhou, 310024 Zhejiang China; 6 Hinda and Arthur Marcus Institute for Aging Research, Hebrew SeniorLife, Boston, USA

**Keywords:** Osteoporosis, Bone density, Cataract

## Abstract

**Summary:**

In two large, ethnically different prospective cohorts from the UK and Hong Kong, osteoporosis was associated with a higher risk of age-related cataracts, particularly in women. Proteomic mediation analysis identified five circulating proteins (MEPE, GDF15, TCN2, CDCP1, SIGLEC1) that may link osteoporosis to cataract development, warranting future mechanistic investigation.

**Purpose:**

Osteoporosis and cataracts are frequently comorbid age-related conditions. This study aims to investigate the association between osteoporosis and the risk of age-related cataracts in cohorts from the United Kingdom (UK) and Hong Kong.

**Methods:**

Prospective cohort studies were conducted in participants aged ≥ 40 years without cataracts at baseline, using data from the UK Biobank (UKB; *n* = 337,952) and the Hong Kong Osteoporosis Study (HKOS; *n* = 4,935), with a median follow-up of 13.5 and 18.5 years, respectively. Incident age-related cataracts were identified using International Classification of Diseases (ICD) codes in each cohort, and the associations of bone mineral density (BMD) and osteoporosis with the risk of age-related cataract were analyzed using Cox proportional hazard models, adjusted for demographics, comorbidities, medications, and blood biomarker levels. We also conducted a mediation analysis to explore potential proteins mediating the relationship.

**Results:**

In UKB, osteoporosis was associated with increased cataract risk (HR, 1.16; 95% CI, 1.08–1.25). In HKOS, participants with BMD T-scores between − 2.5 and − 1 (HR, 1.24; 95% CI, 1.05–1.45) and ≤  − 2.5 (HR, 1.34; 95% CI, 1.09–1.66) had higher cataract risk compared to those with T-scores ≥  − 1. Subgroup analyses suggested the relationship was female-specific in both cohorts. Mediation analysis identified five proteins that may mediate this relationship.

**Conclusion:**

Our findings suggest a female-specific association between osteoporosis and incident age-related cataracts. Integrated care is crucial for patients with these conditions. Timely ophthalmic evaluation and intervention may benefit patients with low BMD.

**Supplementary Information:**

The online version contains supplementary material available at 10.1007/s11657-026-01674-0.

## Introduction

Age-related cataract, the leading cause of blindness in older adults, is characterized by the clouding of the intraocular crystalline lens. Osteoporosis, another age-related disease, commonly coexists with cataracts [1, 2]. Both conditions show increasing prevalence and lead to preventable morbidities in older adults e.g., osteoporotic fractures [3]. A better understanding of the etiological links between them could enhance the management of patients with either or both conditions.

Disruption of calcium homeostasis implicated in both cataractogenesis and osteoporosis. Bone acts as the principal reservoir and regulator of systemic calcium, helping maintain the ionic environment required for lens transparency [4]. Moreover, commonly used anti-osteoporosis therapies—such as bisphosphonates and denosumab—can induce hypocalcemia, particularly in the context of low vitamin D or impaired renal function [5, 6], potentially contributing to cataractogenesis. In addition, cataracts and osteoporosis share molecular pathways such as proteoglycan biosynthesis, which is essential for bone, cornea, and lens health [7, 8]. Defects in proteoglycan-related genes can lead to both osteoporosis and cataracts. For example, mutations in *XYLT2* and *B4GALT7* result in spondyloocular syndrome and Ehlers-Danlos syndrome [9, 10], characterized by both early-onset cataracts and bone fragility. The frequent co-occurrence and shared mechanisms imply a bidirectional relationship between osteoporosis and cataracts. A recent Taiwanese study found that cataract was associated with an increased risk of osteoporosis [11]. However, the association of osteoporosis with the development of cataracts remains understudied.

In this study, we hypothesized that osteoporosis is associated with an increased risk of incident age-related cataracts. We examined this relationship using data from two cohorts – the Hong Kong Osteoporosis Study (HKOS) and the United Kingdom Biobank (UKB). Establishing the relationship could inform integrated management strategies that strengthen ophthalmic surveillance in patients with osteoporosis, reducing vision-related falls and fractures in aging populations.

## Methods

### Study population

HKOS, a cohort study from 1995 to 2010, involved 9,449 Southern Chinese residents aged 12–105 in Hong Kong. Detailed participant profiles and the participant selection process were outlined previously [12]. Clinical, biomedical, bone mineral density (BMD), and genetic data were gathered, along with lifestyle questionnaires. All participants provided informed consent. The participants' health outcomes were tracked longitudinally by linkage to the Clinical Data Analysis and Reporting System (CDARS), a territory-wide electronic health record (EHR) database managed by the Hong Kong Hospital Authority. Ethics approval has been obtained by the Institutional Review Board (IRB) of The University of Hong Kong/HA HKW, Hong Kong Special Administrative Region (UW 23–396).

UKB enrolled around 500,000, predominantly white (94%) participants aged around 40–69 between 2006–2010 in the UK. Participants completed extensive questionnaires, had physical exams, and provided biological samples. It is one of the largest prospective cohorts worldwide with comprehensive ophthalmic data [13]. UKB received ethical approval from a research ethics committee (REC reference 16/NW/0274) and participants provided informed consent.

**Fig. 1:**
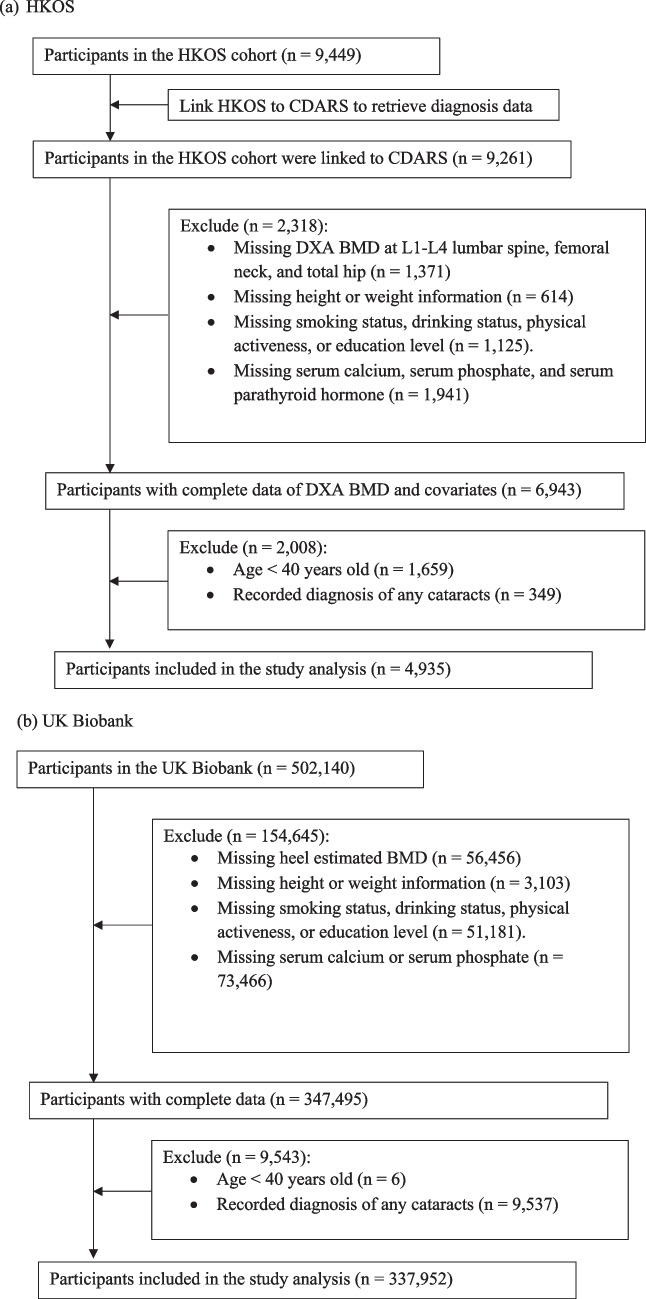
Flowchart of cohort selection

The flowcharts of cohort selection are shown in Fig. 1. In total, we included 4,935 HKOS and 337,952 UKB participants aged ≥ 40 years and without a history of cataracts in the analyses.

### Assessment of BMD and osteoporosis

In HKOS, BMD (g/cm^2^) was measured using a dual-energy X-ray absorptiometer (DXA) (Hologic Inc., Marlborough, MA, USA) at the lumbar spine, femoral neck, and total hip by trained technicians using a standard protocol. Sex-specific BMD T-scores were calculated as the difference between the individual BMD and the young reference BMD of the same sex in standard deviation (SD).

The BMD estimated from quantitative heel ultrasounds (eBMD) and eBMD T-scores were obtained from UKB. As DXA-measured BMD was available for only ~ 10% UKB study population (2014 reassessment), with many key covariates are missing, we opted for eBMD T-scores to maximize sample size and follow-up duration. eBMD exhibited a high correlation with DXA BMD and the ability to predict osteoporotic fractures [14, 15]. Osteoporosis was identified by the “first occurrences” diagnosis records in UKB (i.e. first record in either primary care data, hospital inpatient data, death register records, or self-reported medical condition codes; data category 1712) by the International Classification of Diseases, Tenth Revision (ICD-10) code M80, M81, and M82.

### Ascertainment of outcomes and follow-up

Incident age-related cataracts were identified by the International Classification of Diseases, Ninth Revision (ICD-9) 366.1 in HKOS and ICD-10 H25 in UKB. All participants were followed until the outcome of interest, date of death, or the end of the study (15 January 2024 in HKOS and 31 October 2022 in UKB), whichever came earlier.

### Assessment of covariates

The details of covariates are provided in Supplementary Table 1. Demographic information (height, weight, smoking, drinking habits, physical activity, and education level) was collected via questionnaires in each cohort. Medical histories (diabetes, heart conditions, kidney disease, heart failure, hypertension, dyslipidemia, anemia, and rheumatoid arthritis) were established using ICD-9 (HKOS) and ICD-10 (UKB) codes in the health record systems, or self-reported, or by available blood biomarkers data. In HKOS, baseline steroid and anti-inflammatory medication use were defined by the prescription records in the past year using British National Formulary (BNF) codes (Supplementary Table 1). In the UKB, details of current medication use were first self-reported by a touchscreen questionnaire, followed by verification by trained staff during a verbal interview. Serum calcium and phosphate levels were measured with a Hitachi 747 random access analyzer (Roche Molecular Biochemicals, Mannheim, Germany) in HKOS and by Arsenazo III analysis (calcium) and phosphomolybdate complex analysis (phosphate) on a Beckman Coulter AU5800 in UKB. Parathyroid levels were available only in HKOS, measured by chemiluminescence immunometric assay (Chiron Corporation, MA, USA).

### Statistical analysis

The baseline characteristics of the study cohorts were summarized by sex, with continuous variables summarized as mean and SD and discrete variables presented as frequency (n) and percentages (%). The differences between groups were compared by t-test and χ^2^ test for continuous and categorical variables, respectively.

Cox proportional hazard models were used to determine the association of the DXA-BMD T-scores at the study sites or eBMD T-scores with the risk of incident age-related cataracts by quartiles. Two models were used: a simple-adjusted model with only age, sex, body mass index (BMI), drinking status, smoking status, and physical activity adjusted, and a fully adjusted model with all covariates (baseline education level, medical history, medication, and serum biochemical markers level) adjusted. In addition, we evaluated the association between BMD T-score categories ($\ge$−1, −1 to −2.5, ≤ −2.5) and age-related cataract risk in HKOS and between ICD-identified osteoporosis and age-related cataract risk in UKB. Subgroup analyses by sex were performed in both cohorts.

Multiple sensitivity analyses were performed to test the robustness of our findings. Firstly, we examined the association of continuous DXA-BMD and eBMD T-scores with age-related cataract risk. Additionally, analyses were repeated after further adjustment for serum vitamin D levels in individuals with complete data. The serum estradiol levels were further adjusted in HKOS but not in UKB, as baseline estradiol was measured for most HKOS participants but only a small subset in UKB (mostly pre-menopausal or Hormone Replacement Therapy users [16]). Moreover, cataract events identified within 2 years after enrolment were excluded to mitigate reverse causation.

We also explored whether circulating proteins mediate the association between osteoporosis and age-related cataracts. In a subset of UKB participants (*n* = 54,219), plasma levels of 2,941 protein analytes were measured using the antibody-based Olink Explore 3072 proximity expression assay. Details regarding the assay and data processing have been described previously [17, 18]. Baseline levels of serum proteins as normalized protein expression (NPX) [18] levels were modeled as mediators after adjusting for baseline demographics, including age, sex, BMI, drinking status, smoking status, and physical activity. We first screened proteins using single-mediator analyses, then assessed candidates in a multiple-mediator framework. Only proteins associated with both osteoporosis and incident cataracts were tested. Full details of the mediation analyses are provided in the Supplementary Methods.

All statistical analyses were conducted using R version 4.3.0. (www.R-project.org). All statistical tests were two-sided, and a *P*-value < 0.05 was considered statistically significant except in the simple mediation analysis, where Bonferroni correction was applied. The simple and multiple mediation analyses were based on the R packages “mediation” [19] and “mma” [20] respectively.

## Results

### HKOS

Out of 9,261 HKOS participants linked to CDARS, 4,935 participants remained with complete data after exclusions (Fig. 1a), comprising 3,426 (69.4%) females and 1,509 (30.6%) males, with a mean age of 60.6 years (SD 11.4). Females were more likely to be non-smokers and non-drinkers and tended to have fewer comorbidities and lower BMD T-scores than males (Table 1).

**Table 1:** Demographics of cohorts

	HKOS			UK Biobank		
	Females	Males	P-value	Females	Males	*P*-value
Subjects, n (%)	3426 (69.4)	1509 (30.6)		179407 (53.1)	158545 (46.9)	
Age, mean ± SD	59.0 (11.1)	64.2 (11.1)	< 0.001	56.0 (8.0)	56.5 (8.2)	< 0.001
BMI, mean ± SD	23.4 (3.6)	23.4 (3.2)	0.466	26.0 (5.1)	27.8 (4.2)	< 0.001
Drinking Status, *n* (%)			< 0.001			< 0.001
Non-drinker	3259 (95.1)	1097 (72.7)		9090 (5.1)	3784 (2.4)	
Ex-drinker	51 (1.5)	155 (10.3)		6119 (3.4)	5172 (3.3)	
Current-drinker	116 (3.4)	257 (17.0)		164198 (91.5)	149589 (94.4)	
Smoking Status, *n* (%)			< 0.001			< 0.001
Non-smoker	3292 (96.1)	990 (65.6)		107399 (59.9)	78747 (49.7)	
Ex-smoker	78 (2.3)	346 (22.9)		56893 (31.7)	60968 (38.5)	
Current-smoker	56 (1.6)	173 (11.5)		15115 (8.4)	18830 (11.9)	
Physically Active, *n* (%)	1700 (49.6)	861 (57.1)	< 0.001	89228 (49.7)	84615 (53.4)	< 0.001
Education level, *n* (%)			< 0.001			
No	584 (17.0)	93 (6.2)		-	-	< 0.001
Primary School	1067 (31.1)	362 (24.0)		25671 (14.3)	23987 (15.1)	
Secondary School	1264 (36.9)	642 (42.5)		46877 (26.1)	30269 (19.1)	
Post-secondary education (including college or university)	511 (14.9)	412 (27.3)		106859 (59.6)	104289 (65.8)	
Diabetes, *n* (%)	292 (8.5)	183 (12.1)	< 0.001	5123 (2.9)	9131 (5.8)	< 0.001
Coronary Heart Disease, *n* (%)	120 (3.5)	170 (11.3)	< 0.001	4726 (2.6)	11647 (7.3)	< 0.001
Chronic Kidney Disease, *n* (%)	77 (2.2)	42 (2.8)	0.303	3817 (2.1)	3564 (2.2)	0.018
Heart Failure, *n* (%)	32 (0.9)	26 (1.7)	0.026	356 (0.2)	1214 (0.8)	< 0.001
Hypertension, *n* (%)	617 (18.0)	450 (29.8)	< 0.001	38984 (21.7)	47749 (30.1)	< 0.001
Dyslipidemia, *n* (%)	193 (5.6)	172 (11.4)	< 0.001	19326 (10.8)	29078 (18.3)	< 0.001
Anemia, *n* (%)	153 (4.5)	49 (3.2)	0.056	10187 (5.7)	3434 (2.2)	< 0.001
Rheumatoid Arthritis, n (%)	25 (0.7)	10 (0.7)	0.941	2763 (1.5)	1334 (0.8)	< 0.001
Use of Steroids^a^, *n* (%)	43 (1.3)	36 (2.4)	0.005	4996 (2.8)	4121 (2.6)	0.001
Use of Anti-inflammatory Drugs^a^, *n* (%)	487 (14.2)	177 (11.7)	0.021	32327 (18.0)	20292 (12.8)	< 0.001
Serum Calcium (mmol/L), mean ± SD	2.39 (0.09)	2.38 (0.09)	< 0.001	2.39 (0.10)	2.37 (0.09)	< 0.001
Serum Phosphate (mmol/L), mean ± SD	1.14 (0.15)	1.06 (0.15)	< 0.001	1.20 (0.15)	1.12 (0.16)	< 0.001
Serum Parathyroid Hormones (pmol/L), mean ± SD	3.75 (1.91)	4.35 (2.16)	< 0.001	-	-	-
L1—L4 Lumbar Spine BMD T-score, mean ± SD	−1.3 (1.3)	−0.3 (1.3)	< 0.001	-	-	
Femoral Neck BMD T-score, mean ± SD	−1.3 (1.3)	−0.9 (0.8)	< 0.001	-	-	
Total Hip BMD T-score, mean ± SD	−1.1 (1.4)	−0.7 (1.0)	< 0.001	-	-	
Heel estimated BMD T-score, mean ± SD	-	-		−0.54 (1.10)	−0.04 (1.37)	< 0.001

^a^The drug use was the prescription in the past year in HKOS; self-reporting current use in UKB

After a median follow-up of 18.5 years (interquartile range [IQR], 12.3 to 20.8 years), 1,065 incident cases of age-related cataracts were identified, and a total of 931 deaths were censored. The total follow-up time was 79,446.6 person-years, and the incidence rate was 13.4 per 1,000 person-years. The highest quartile of DXA-measured BMD T-scores at lumbar spine L1-L4, femoral neck, and total hip were significantly associated with a lower risk of age-related cataracts relative to the lowest quartile in all models (Table 2), with a fully adjusted HR of 0.77 (95% CI, 0.62 to 0.94), 0.77 (95% CI, 0.62 to 0.97), and 0.74 (95% CI, 0.59 to 0.92), respectively. In addition, compared to those with BMD T-scores $\ge$ −1, those with −2.5 T$<$-scores $<$ −1 (HR, 1.24; 95% CI, 1.05 to 1.45) and those with T-scores $\le$ −2.5 (HR, 1.34; 95% CI, 1.09 to 1.66) had a significantly higher risk of developing age-related cataracts (Table 3). The subgroup analysis by sex indicated that the relationship is female-specific with HR of 1.38 (95% CI, 1.08 to 1.75) and 1.61 (95% CI, 1.22 to 2.11) in those with −2.5 T$<$-scores $<$ −1 and T-scores $\le$ −2.5, respectively.

**Table 2:** Association between DXA-BMD T-scores and estimated BMD T-scores by quartiles and the risk of incident age-related cataracts in HKOS and UK Biobank

	Quartile 1	Quartile 2		Quartile 3		Quartile 4	
BMD T-scores in HKOS	HR (ref)	HR (95% CI)	*P*-value	HR (95% CI)	*P*-value	HR (95% CI)	*P*-value
^a^Model 1							
Lumbar spine L1-L4	1	0.93 (0.79, 1.09)	0.363	0.85 (0.70, 1.02)	0.080	0.77 (0.62, 0.94)	0.011
Femoral neck	1	0.99 (0.84, 1.18)	0.943	1.00 (0.83, 1.20)	0.985	0.76 (0.61, 0.95)	0.017
Total hip	1	1.00 (0.85, 1.18)	0.954	0.98 (0.81, 1.18)	0.825	0.72 (0.58, 0.90)	0.005
^b^Model 2							
Lumbar spine L1-L4	1	0.93 (0.79, 1.09)	0.367	0.84 (0.69, 1.01)	0.067	0.77 (0.62, 0.94)	0.012
Femoral neck	1	1.00 (0.84, 1.19)	0.991	1.00 (0.83, 1.20)	0.981	0.77 (0.62, 0.97)	0.027
Total hip	1	1.01 (0.85, 1.20)	0.906	1.00 (0.83, 1.20)	0.984	0.74 (0.59, 0.92)	0.008
eBMD T-scores in UK Biobank							
^a^Model 1	1	0.95 (0.91, 0.98)	0.003	0.96 (0.92, 0.99)	0.021	0.96 (0.92, 1.00)	0.030
^c^Model 2	1	0.95 (0.92, 0.98)	0.004	0.96 (0.93, 1.00)	0.032	0.96 (0.92, 1.00)	0.045

^a^Model 1: Adjusted for age, sex, BMI, smoking status, drinking status, and physical activeness,

^b^Model 2: Adjusted for age, sex, BMI, smoking status, drinking status, physical activeness, education level, the medical history of diabetes, coronary heart disease, chronic kidney disease, heart failure, hypertension, dyslipidemia, anemia, rheumatoid arthritis, the baseline medication use of steroids and anti-inflammatory drugs in the past year, and serum calcium, serum phosphate, and serum parathyroid level

^c^Model 2: Adjusted for age, sex, BMI, smoking status, drinking status, physical activeness, education level, the medical history of diabetes, coronary heart disease, chronic kidney disease, heart failure, hypertension, dyslipidemia, anemia, rheumatoid arthritis, the self-reported baseline medication use of steroids and anti-inflammatory drugs, and serum calcium and serum phosphate level

**Table 3:** Association between DXA BMD T-score categories and the risk of incident age-related cataract in HKOS

	BMD T-scores $\ge -$ 1^a^		$-2.5<$ BMD T-scores $<-1$ ^a^			BMD T-scores $\le -2.5$ ^a^		
	*n*	HR (ref)	*n*	HR^b^ (95% CI)	*P*-value	*n*	HR^b^ (95% CI)	*P*-value
All individuals	1741	1	2160	1.24 (1.05, 1.45)	0.010	1034	1.34 (1.09, 1.66)	0.007
By sex								
Females	991	1	1489	1.38 (1.08, 1.75)	0.009	946	1.61 (1.22, 2.11)	0.001
Males	750	1	671	1.13 (0.90, 1.42)	0.298	88	0.70 (0.38, 1.30)	0.259

^a^At any sites of L1-4 lumbar spine, femoral neck, and total hip

^b^Adjusted for age, sex, BMI, smoking status, drinking status, physical activeness, education level, medical history of diabetes, coronary heart disease, chronic kidney disease, heart failure, hypertension, dyslipidemia, anemia, rheumatoid arthritis, baseline medication use of steroids, and anti-inflammatory drugs in the past year, and serum calcium, serum phosphate, and serum parathyroid hormone level

When modelling BMD T-scores continuously, a significant inverse relationship with incident age-related cataracts was observed at the L1-L4 lumbar spine (Supplementary Table 2). Consistent associations were observed in the sensitivity analyses, adjusting for serum vitamin D level (Supplementary Table 3) and serum estradiol level (Supplementary Table 4), and after excluding cataract cases identified within 2 years of enrolment (Supplementary Table 5). Notably, the association was also observed in postmenopausal women after adjusting for estradiol level (Supplementary Table 4).

### UKB

From 502,140 UKB participants, 337,952 participants with complete data were included in the study after exclusions (Fig. 1b). The cohort comprised 179,407 (53.1%) females and 158,545 (46.9%) males with a younger mean age (56.2 [SD 8.1]) compared to the HKOS (Table 1). In addition, UKB had a much higher proportion of current drinkers and individuals who had undertaken post-secondary education than HKOS (Table 1).

The total follow-up of UKB was 4,386,020 person-years, with a median follow-up time of 13.5 years (IQR, 12.7 to 14.2 years). 22,020 incident cases of age-related cataracts were identified, and a total of 26,151 deaths were censored. There were 6,527 participants with diagnosed osteoporosis. The second, third, and fourth quartiles of eBMD were all associated with a lower risk of age-related cataracts compared to the lowest quartile, with fully adjusted HRs of 0.95 (95% CI, 0.92 to 0.98), 0.96 (95% CI, 0.93 to 1.00), and 0.96 (95% CI, 0.92 to 1.00) (Table 2). Osteoporosis was associated with a significantly increased risk of age-related cataracts in both the simple-adjusted model (HR, 1.18; 95% CI, 1.10 to 1.27) and the fully-adjusted model (HR, 1.16; 95% CI, 1.08 to 1.25) (Table 4). A female-specific relationship between osteoporosis and age-related cataracts was also evident in UKB (HR, 1.16; 95% CI, 1.08 to 1.26). Consistent results were observed after adjusting for serum vitamin D levels (Supplementary Table 6) and excluding cataract cases identified within 2 years of enrolment (Supplementary Table 7).

**Table 4:** Association between the diagnosis of osteoporosis and the risk of incident age-related cataracts in the UK Biobank

	No diagnosis of osteoporosis		Diagnosis of Osteoporosis				
				Model 1^a^		Model 2^b^	
	Case number/Total number	HR (ref)	Case number/Total number	HR (95% CI)	*P*-value	HR (95% CI)	*P*-value
All individuals	21226/331425	1	794/6527	1.18 (1.10, 1.27)	< 0.001	1.16 (1.08, 1.25)	< 0.001
By sex							
Females	12100/173921	1	704/5486	1.18 (1.09, 1.27)	< 0.001	1.16 (1.08, 1.26)	< 0.001
Males	9126/157504	1	90/1041	1.15 (0.93, 1.41)	0.196	1.06 (0.86, 1.31)	0.563

^a^Model 1: Adjusted for age, sex, BMI, smoking status, drinking status, and physical activeness

^b^Model 2: Adjusted for age, sex, BMI, smoking status, drinking status, physical activeness, education level, the medical history of diabetes, coronary heart disease, chronic kidney disease, heart failure, hypertension, dyslipidemia, anemia, rheumatoid arthritis, the self-reported baseline medication use of steroids, anti-inflammatory drugs, and serum calcium and serum phosphate level

### Mediation analysis

Of 2,931 circulating proteins with measurements available, 283 were associated with both osteoporosis and incident age-related cataracts and assessed for potential mediation. Of these, 58 proteins had a statistically significant average causal mediated effect (ACME; the effect of the mediator alone) in the simple mediation analysis after correction for multiple testing (*p* < 0.05/283) and were considered candidate mediators of the relationship between osteoporosis and cataracts (Supplementary Table 8).

In the multiple mediation model, only 9 proteins were found to be significantly associated with both osteoporosis and age-related cataracts when assessing all candidate proteins simultaneously. Among these, 5 proteins—Matrix Extracellular Phosphoglycoprotein (MEPE), Growth Differentiation Factor 15 (GDF15), Transcobalamin 2 (TCN2), CUB Domain-Containing Protein 1 (CDCP1), and Sialoadhesin (SIGLEC1)—demonstrated a statistically significant individual ACME (Supplementary Table 9).

## Discussion

In this large, prospective cohort study on HKOS and UKB, we consistently observed that osteoporosis is associated with a higher risk of incident age-related cataracts. Subgroup analysis suggests the relationship is female-specific. Moreover, five circulating proteins—MEPE, GDF15, TCN2, CDCP1, and SIGLEC1 —were identified as potential mediators of the relationship, warranting future mechanistic investigations.

Prior studies predominantly address the unidirectional influence of cataracts on osteoporosis. Increased osteoporosis has been shown in cataract surgery recipients among women of all age groups and in men > 75 years [1]. In addition, the diagnosis of cataracts was associated with an increased risk of developing osteoporosis or fractures, and undergoing cataract surgery may mitigate these risks [11]. Our study is the first to show that low BMD and the diagnosis of osteoporosis are associated with increased risk of age-related cataracts, supporting a bidirectional relationship. Notably, a previous Korean study found an association between osteoporosis and age-related macular degeneration (AMD) in females but not with cataracts [21]. The inconsistency may be attributed to its cross-sectional design and potential selection biases in DXA measurements and ophthalmic examinations. In contrast, our study leverages two large, ethnically different cohorts with an extended follow-up, providing a more robust evaluation of osteoporosis as a risk factor for subsequent cataract.

Calcium imbalance is thought to be a key factor in the association between osteoporosis and cataracts, given calcium homeostasis is crucial for bone health and the development of cataracts [1, 22]. Despite adjusting for serum calcium, measurements at a single time-point may not adequately capture chronic mineral dysregulation. In contrast, low BMD could serve as a more indicative marker of a chronic disorder of calcium homeostasis and mineralization, thereby associated with the risk of developing cataracts. Vitamin D deficiency and inflammation are additional shared factors that could underlie the bidirectional relationship [23, 24]. Adjusting for vitamin D did not attenuate the association (Supplementary Table 3 and 6), but residual confounding remains possible given unmeasured or hard-to-measured factors like aging, systemic inflammatory status, and oxidative stress pathways. These common factors may drive co-occurrence rather than a strict causal effect of osteoporosis on cataracts. Whether low BMD and cataracts primarily reflect shared aging biology warrants further study.

To generate mechanistic hypotheses, we performed mediation analysis and identified five circulating proteins (MEPE, GDF15, TCN2, CDCP1, and SIGLEC1) as potential mediators. These proteins participate in pathways relevant to mineral metabolism, inflammation, and bone remodeling. For example, MEPE negatively regulates the mineralization process and is a candidate phosphatonin and affects phosphate homeostasis [25, 26], raising the possibility that osteoporosis-related MEPE dysregulation affects cataract development through mineral imbalance. GDF15, a hormone in the TGF-β superfamily, implicated in both bone metabolism and inflammatory pathways [27, 28] and may influence eBMD [29]. TCN2 is a transporter of vitamin B-12, a nutrient implicated in both bone and eye pathology, whereas the roles of CDCP1 and SIGLEC1 in cataract development remain unclear. Accordingly, these mediation results should be considered exploratory; causal inference cannot be established from observational data and will require replication, genetic analyses, and experimental studies.

Moreover, our study and past research both suggest that the relationship between osteoporosis and cataracts is more evident in females than in males. This may partly reflect the lower incidence of both conditions in males than females, but biological differences are also plausible. Estrogen decline is a key driver for osteoporosis in females and has been linked to higher cataract risk [30, 31]. Notably, the female-specific association persisted after adjusting for serum estradiol in HKOS and among postmenopausal women—who constitute the majority of osteoporosis cases and have relatively stable, low estrogen levels (Supplementary Table 4). These results suggest mechanisms beyond estrogen deficiency. Additional female-specific factors not captured in our data (e.g., reproductive history, hormone therapy, surgical menopause), and sex differences in oxidative stress may contribute to the observed association and warrant further investigation.

The study has important clinical implications. In osteoporotic patients, the higher risk of cataracts may amplify their fracture risk through vision impairment and a higher likelihood of falls [3]. Considering the substantial mortality associated with fractures in older adults, it is crucial to effectively manage both osteoporosis and cataracts to mitigate the risk of fractures. Research has indicated that cataract patients who underwent cataract surgeries experienced a 42% reduction in the risk of developing osteoporosis or experiencing fractures [11] and another showed lower odds of hip fracture within 1 year postoperatively in US Medicare beneficiaries aged 65 and older [32]. Together, these findings support integrated management of bone and eye health in older adults, particularly those with low BMD, e.g. screening for visual impairment, timely cataract management, and optimizing fracture prevention.

This study has several strengths, including its longitudinal design, large sample size, extended follow-up, replication across two ethnically distinct cohorts, and consistent results in sensitivity analyses, all supporting the robustness of the findings. Nevertheless, several limitations warrant consideration. First, age-related cataracts were identified using hospital diagnosis codes, which may miss some early-stage cataract cases and fail to capture the true onset date or severity of cataracts, potentially underestimating incidence. However, this misclassification would more likely underestimate the observed relationship rather than overestimate it. Second, the BMD-cataract risk relationship may be nonlinear; however, higher BMD quartiles consistently demonstrated reduced risk. Third, in UKB, we used heel eBMD rather than DXA BMD due to greater data availability. Heel eBMD is less precise than central DXA BMD (lumbar spine and femoral neck) and World Health Organization’s T-score criteria for diagnosing osteoporosis are not applicable to eBMD [15]; therefore, we analyzed ICD-coded osteoporosis as the exposure in UKB. Fourth, generalizability beyond the Hong Kong Chinese and UK populations is uncertain. Fifth, the results are largely driven by females, while male subgroup analyses did not show statistically significant associations. Further studies with larger sample size of older men are warranted to assess the association in males. Although the overall association appeared significant, it should not be generalized without considering this sex-specific pattern. Finally, residual confounding may persist given the observational design, despite adjusting for many key covariates. Moreover, the proteins examined as potential mediators may also act as confounders (Directed Acyclic Graph [DAG] shown in Supplementary Fig. 1), influencing both osteoporosis and cataract development. As such, the mediation analysis should be considered exploratory, with its findings interpreted with caution.

## Conclusions

In conclusion, osteoporosis is associated with a higher risk of age-related cataracts, especially in females. Timely ophthalmic evaluation and intervention may benefit patients with low BMD.

## Supplementary Information

Below is the link to the electronic supplementary material.

ESM 1 (DOCX 157 KB)

ESM 2 (PDF 87.1 KB)

## Data Availability

The HKOS dataset used in the current study cannot be shared due to The Personal Data (Privacy) Ordinance (Cap. 486) in Hong Kong as stated in the patient consent form. For all requests regarding data, please contact the corresponding author at lung1212@hku.hk. Data from the UK Biobank is available to approved researchers through the UK Biobank Access Management System https://www.ukbiobank.ac.uk/.
