# Supplementary material for: Sex-specific association between osteoporosis and cataracts: UK Biobank and Hong Kong Osteoporosis Study
Source: Arch Osteoporos. 2026 Feb 24;21(1):44. doi: 10.1007/s11657-026-01674-0 (PMC12932269; doi:10.1007/s11657-026-01674-0)
Supplement: Supplementary file 1 — ESM 1 (DOCX 157 KB) [file 11657_2026_1674_MOESM1_ESM.docx]

**Sex-specific association between osteoporosis and cataracts: UK Biobank and Hong Kong Osteoporosis Study**

Xiaowen Zhang*, Suhas Krishnamoorthy*, Kelvin KL Chong, Jonathan KL Mak, Kathryn Choon-Beng Tan, Annie Wai-Chee Kung, Ian Chi-Kei Wong, Hou-Feng Zheng, Ching-Lung Cheung

*Co-first author

**Supplementary Materials**

| **Name** | **Title** | **Page number** |
| --- | --- | --- |
| Supplementary Methods | Mediation analysis | 2 |
| Supplementary Table 1 | Details of covariates and ICD-9 and ICD-10 codes and BNF used. | 3-4 |
| Supplementary Table 2 | Cox proportional hazard models examining the relationship between the BMD T-scores and the risk of incident age-related cataracts. | 5 |
| Supplementary Table 3 | Association between osteoporosis status (normal, osteopenia, and osteoporosis) categorized by DXA BMD T-scores and the risk of incident age-related cataract in HKOS further adjusted for serum 25(OH)D level (n = 4,051) | 6 |
| Supplementary Table 4 | Association between osteoporosis status (normal, osteopenia, and osteoporosis) categorized by DXA BMD T-scores and the risk of incident age-related cataract in HKOS further adjusted for serum estradiol level (n = 4,826) | 7 |
| Supplementary Table 5 | Association between osteoporosis status (normal, osteopenia, and osteoporosis) categorized by DXA BMD T-scores and the risk of incident age-related cataract in HKOS after excluding cataract cases identified within 2 years of enrolment (n = 4,840) | 8 |
| Supplementary Table 6 | Association between the diagnosis of osteoporosis and the risk of incident age-related cataracts in the UK Biobank further adjusted for serum vitamin D level (n = 322,608) | 9 |
| Supplementary Table 7 | Association between the diagnosis of osteoporosis and the risk of incident age-related cataracts in the UK Biobank after excluding cataract cases identified within 2 years of enrolment (n = 337,298) | 10 |
| Supplementary Table 8 | Results of the mediation analysis in the UK Biobank. | Separate PDF file |
| Supplementary Table 9 | 9 proteins found to be significantly associated with both osteoporosis and age-related cataracts. | 11 |
| Supplementary Figure 1 | Directed Acyclic Graph (DAG) illustrating the multiple mediation model. | 12 |

**Supplementary Methods**

Mediation analysis

Given the complexity of protein interactions, we initially employed a simple mediation analysis to identify potential single mediators among the complete set of available proteins. Subsequently, we utilized a multiple mediation analysis to explore these candidate proteins within a multiple mediation framework. This approach allowed us to discern proteins that are likely to act as mediators in the context of other interacting proteins.

1. Simple mediation analysis

In the simple mediation analysis, the association of osteoporosis with NPX levels was assessed using linear regression, while that of NPX levels and osteoporosis with age-related cataracts was assessed using a parametric survival model with Weibull distribution. ^1^ These two models were used to estimate the total effect of the exposure on the outcome and then dissect it into the indirect effect (i.e. through the protein) and the direct effect (i.e. through pathways independent of the protein). ^2^ The proportion of the effect that is mediated by osteoporosis-related proteins was also evaluated. Mediation analysis works under the assumption of sequential ignorability, or no unmeasured confounding. Mediation analysis also assumes a temporal sequence: exposure → mediator → outcome. Our exposure–mediator data are cross-sectional, however, osteoporosis develops over years, so most cases likely preceded protein measurement, supporting directionality despite possible reverse causation or residual confounding. For the mediator–outcome link, we used longitudinal Cox models, preserving temporal order between protein levels and incident cataracts. Findings are presented as the Proportion Mediated (PM) in percentage and as Average Causal Mediated Effect (ACME), Average Direct Effect (ADE), and Total Effect (TE) estimates. Quasi-Bayesian confidence intervals were constructed for the estimated effects with 1000 simulations. Proteins exhibiting a statistically significant mediated effect after Bonferroni correction were considered as candidate mediating proteins.

1. Multiple mediation analysis

To further investigate the mediating roles of proteins, we used the “mma” R package^3^ to assess multiple potential mediators simultaneously within a comprehensive mediation model. Similar to our initial analysis, the model was designed to partition the total effect of exposure on the outcome into both direct and indirect effects mediated by the selected proteins. However, a Cox proportional hazards model was used here to estimate the effect of the mediating proteins on the outcome, as Cox is compatible with the “mma” package and generally preferred in clinical studies due to its greater interpretability. ^4^ The 95% confidence intervals were calculated using 1,000 bootstrap resamples. Notably, the word “effect” is used as the notion in the mediation analysis; however, causal effects cannot be inferred due to the observational study's nature.

1. VanderWeele TJ. Causal mediation analysis with survival data. Epidemiology 2011; 22(4): 582-5.

2. Imai K, Keele L, Yamamoto T. Identification, Inference and Sensitivity Analysis for Causal Mediation Effects. Statistical Science 2010; 25(1): 51-71, 21.

3. Yu Q, Wu X, Li B, Scribner RA. Multiple mediation analysis with survival outcomes: With an application to explore racial disparity in breast cancer survival. Stat Med 2019; 38(3): 398-412.

4. Nardi A, Schemper M. Comparing Cox and parametric models in clinical studies. Stat Med 2003; 22(23): 3597-610.

Supplementary Table 1. Details of covariates and ICD-9 and ICD-10 codes and BNF used.

| **Demographics** | **HKOS** | **UK Biobank** |
| --- | --- | --- |
| Sex | Males; females | Males; females |
| Age | Year on index date | Year on index date |
| BMI | kg/m^2^ | kg/m^2^ |
| Smoking status | Non-smokers (smoked < 20 packs of cigarettes in their lifetime); ex-smokers (ceased smoking for at least 1 year); current smokers | Based on response to touchscreen questionnaire.  Current smokers did not answer no to “Do you smoke tobacco now?”  Previous smokers answered either “On all or most days” or “Occaisonally” to the question “In the past, how often have you smoked tobacco” |
| Drinking status | Non-drinkers (had < 12 alcoholic drinks in their lifetime); ex-drinkers (ceased drinking for at least 1 year); current drinkers | Based on response to touchscreen questionnaire. Current drinkers were those who did not answer “Never” to “About how often do you drink alcohol?".  Those who answered “Never” were given the question “Did you previously drink alcohol”. Those who answered yes were considered previous drinkers. |
| Physically active | Active (> 180 minutes a week); not active (<= 180 minutes a week) | Active (moderate physical activity >= 5 days a week or vigorous physical activity >= 3 days a week).^5^ |
| Education level | No; primary school; secondary school; University and college | Educational qualification was mapped to an International Standard Classification of Education (ISCED) category as shown previously.^6^ ISCED category was further mapped to:  Post-secondary (ISCED 4 OR 5)  Secondary (ISCED 2 OR 3)  Primary (ISCED 1) |
| **Diagnosis** | **ICD-9** | **ICD-10** |
| Cataracts | 366 |  |
| Age-related (senile) cataracts | 366.1 | H25 |
| Other cataracts |  | H26, H28 |
| Infantile, juvenile, and presenile cataract | 366.0 |  |
| Traumatic cataract | 366.2 |  |
| Cataract secondary to ocular disorders | 366.3 |  |
| Cataract associated with other disorders | 366.4 |  |
| After-cataract | 366.5 |  |
| Other and unspecified cataracts | 366.8, 366.9 |  |
| **Comorbidities** | Self-reported or identified by ICD-9 (or blood biomarkers) | Self-reported or identified by ICD-10 (or blood biomarkers) |
| Diabetes | 250 | E10, E11, or random glucose >=11.1mmol/l or HbA1c >=48mmol/mol |
| Coronary heart disease | 410-414, 429.2, 429.71, 429.79 | I20-I25 |
| Chronic kidney disease | 403, 404, 582, 585, 586, 590.0, V56, or eGFR <60 mL/min/1.73m^2^ | N28 or eGFR <60 mL/min/1.73m^2^ |
| Heart failure | 398.91, 402.01, 402.11, 402.91, 404.01, 404.03, 404.11, 404.13, 404.91, 404.93, 428 | I50 |
| Hypertension | 401, 402, 403, 405 | I10-I12, I15 |
| Dyslipidemia | 272 | E78 |
| Anemia | 280-285, or baseline hemoglobin levels <12.0 g/dL in women and <13.0 g/dL in men | D50-D64 |
| Rheumatoid arthritis | 446.5, 710, 714, 714.8, 725 | M05, M06 |
|  |  |  |
| **Baseline medication in the past year** | **BNF** | **Self-reported in verbal interview** |
| Steroids | 6.3 | Use of any drugs listed in BNF 6.3 |
| Anti-inflammatory drugs | 10.1.1 | Use of any drugs listed in BNF 10.1.1 |

5. Pate RR, Pratt M, Blair SN, et al. Physical activity and public health. A recommendation from the Centers for Disease Control and Prevention and the American College of Sports Medicine. *Jama* 1995;273(5):402-7. doi: 10.1001/jama.273.5.402

6. Lee JJ, Wedow R, Okbay A, et al. Gene discovery and polygenic prediction from a genome-wide association study of educational attainment in 1.1 million individuals. *Nature Genetics* 2018;50(8):1112-21. doi: 10.1038/s41588-018-0147-3

Supplementary Table 2. Cox proportional hazard models examining the relationship between the BMD T-scores and the risk of incident age-related cataracts.

|  | **Model 1^a^** | | **Model 2^b^** | |
| --- | --- | --- | --- | --- |
|  | **HR (95% CI)** | **P-value** | **HR (95% CI)** | **P-value** |
| BMD T-scores at |  |  |  |  |
| L1-L4 Lumbar spine | 0.94 (0.89, 0.99) | 0.023 | 0.94 (0.89, 0.99) | 0.027 |
| Femoral neck | 0.93 (0.87, 1.00) | 0.058 | 0.94 (0.87, 1.01) | 0.081 |
| Total hip | 0.94 (0.89, 1.00) | 0.066 | 0.95 (0.89, 1.01) | 0.115 |
| eBMD T-scores | 0.99 (0.98, 1.00) | 0.064 | 0.99 (0.98, 1.00) | 0.095 |

^a^Model 1: Adjusted for age, sex, and BMI, smoking status, drinking status, and physical activeness.

^b^Model 2: Adjusted for age, sex, BMI, smoking status, drinking status, physical activeness, education level, the medical history of diabetes, coronary heart disease, chronic kidney disease, heart failure, hypertension, dyslipidemia, anemia, rheumatoid arthritis, the baseline medication use of steroids and anti-inflammatory drugs, and serum calcium, serum phosphate, and serum parathyroid level (only in HKOS).

Supplementary Table 3. Association between osteoporosis status (normal, osteopenia, and osteoporosis) categorized by DXA BMD T-scores and the risk of incident age-related cataract in HKOS further adjusted for serum 25(OH)D level (n = 4,051)

|  | **BMD T-scores** $\boldsymbol{\geq-}$**1^a^** | |  | $\boldsymbol{-}\mathbf{2.5<}$**BMD T-scores** $\mathbf{<-1}$**^a^** | | |  | **BMD T-scores** $\boldsymbol{\leq-2.5}$**^a^** | | |
| --- | --- | --- | --- | --- | --- | --- | --- | --- | --- | --- |
|  | n | HR (ref) |  | n | HR^b^ (95% CI) | P-value |  | n | HR^b^ (95% CI) | P-value |
| All individuals | 1476 | 1 |  | 1775 | 1.19 (1.00, 1.41) | 0.049 |  | 800 | 1.30 (1.04, 1.64) | 0.023 |
| By sex |  |  |  |  |  |  |  |  |  |  |
| Females | 802 | 1 |  | 1178 | 1.29 (1.00, 1.66) | 0.050 |  | 728 | 1.56 (1.16, 2.09) | 0.003 |
| Males | 674 | 1 |  | 597 | 1.10 (0.87, 1.40) | 0.433 |  | 72 | 0.61 (0.31, 1.22) | 0.161 |

^a^At any sites of L1-4 lumbar spine, femoral neck, and total hip.

^b^Adjusted for age, sex, BMI, smoking status, drinking status, physical activeness, education level, medical history of diabetes, coronary heart disease, chronic kidney disease, heart failure, hypertension, dyslipidemia, anemia, rheumatoid arthritis, baseline medication use of steroids and anti-inflammatory drugs in the past year, and serum calcium, serum phosphate, serum parathyroid hormone, and serum 25(OH)D level.

Supplementary Table 4. Association between osteoporosis status (normal, osteopenia, and osteoporosis) categorized by DXA BMD T-scores and the risk of incident age-related cataract in HKOS further adjusted for serum estradiol level (n = 4,826)

|  | **BMD T-scores** $\boldsymbol{\geq-}$**1^a^** | |  | $\boldsymbol{-}\mathbf{2.5<}$**BMD T-scores** $\mathbf{<-1}$**^a^** | | |  | **BMD T-scores** $\boldsymbol{\leq-2.5}$**^a^** | | |
| --- | --- | --- | --- | --- | --- | --- | --- | --- | --- | --- |
|  | n | HR (ref) |  | n | HR^b^ (95% CI) | P-value |  | n | HR^b^ (95% CI) | P-value |
| All individuals | 1694 | 1 |  | 2119 | 1.21 (1.03, 1.43) | 0.019 |  | 1013 | 1.28 (1.03, 1.58) | 0.027 |
| By sex |  |  |  |  |  |  |  |  |  |  |
| Females | 945 | 1 |  | 1456 | 1.30 (1.02, 1.66) | 0.033 |  | 927 | 1.49 (1.13, 1.96) | 0.005 |
| Post-menopausal^c^ | 481 | 1 |  | 1219 | 1.21 (0.94, 1.56) | 0.134 |  | 912 | 1.38 (1.04, 1.83) | 0.024 |
| Males | 749 | 1 |  | 663 | 1.15 (0.91, 1.45) | 0.235 |  | 86 | 0.67 (0.36, 1.27) | 0.223 |

^a^At any sites of L1-4 lumbar spine, femoral neck, and total hip.

^b^Adjusted for age, sex, BMI, smoking status, drinking status, physical activeness, education level, medical history of diabetes, coronary heart disease, chronic kidney disease, heart failure, hypertension, dyslipidemia, anemia, rheumatoid arthritis, baseline medication use of steroid and anti-inflammatory drugs in the past year, and serum calcium, serum phosphate, serum parathyroid hormone, and serum estradiol level.

^c^Only among those with menopausal status recorded, the subset of post-menopausal females (n = 2,612).

Supplementary Table 5. Association between osteoporosis status (normal, osteopenia, and osteoporosis) categorized by DXA BMD T-scores and the risk of incident age-related cataract in HKOS after excluding cataract cases identified within 2 years of enrolment (n = 4,840)

|  | **BMD T-scores** $\boldsymbol{\geq-}$**1^a^** | |  | $\boldsymbol{-}\mathbf{2.5<}$**BMD T-scores** $\mathbf{<-1}$**^a^** | | |  | **BMD T-scores** $\boldsymbol{\leq-2.5}$**^a^** | | |
| --- | --- | --- | --- | --- | --- | --- | --- | --- | --- | --- |
|  | n | HR (ref) |  | n | HR^b^ (95% CI) | P-value |  | n | HR^b^ (95% CI) | P-value |
| All individuals | 1723 | 1 |  | 2121 | 1.26 (1.07, 1.49) | 0.006 |  | 996 | 1.40 (1.12, 1.74) | 0.003 |
| By sex |  |  |  |  |  |  |  |  |  |  |
| Females | 987 | 1 |  | 1467 | 1.39 (1.08, 1.77) | 0.009 |  | 912 | 1.66 (1.25, 2.20) | <0.001 |
| Males | 736 | 1 |  | 654 | 1.18 (0.93, 1.50) | 0.181 |  | 84 | 0.65 (0.32, 1.32) | 0.230 |

^a^At any sites of L1-4 lumbar spine, femoral neck, and total hip.

^b^Adjusted for age, sex, BMI, smoking status, drinking status, physical activeness, education level, medical history of diabetes, coronary heart disease, chronic kidney disease, heart failure, hypertension, dyslipidemia, anemia, rheumatoid arthritis, baseline medication use of steroid and anti-inflammatory drugs in the past year, and serum calcium, serum phosphate, and serum parathyroid hormone level.

Supplementary Table 6. Association between the diagnosis of osteoporosis and the risk of incident age-related cataracts in the UK Biobank further adjusted for serum vitamin D level (n = 322,608)

|  | **No diagnosis of osteoporosis** | | **Diagnosis of Osteoporosis** | | |
| --- | --- | --- | --- | --- | --- |
|  | Case number/ total number | HR (ref) | Case number/ total number | HR^a^ (95% CI) | P-value |
| All individuals | 20114/316448 | 1 | 743/6160 | 1.16 (1.08, 1.25) | <0.001 |
| By sex |  |  |  |  |  |
| Females | 11232/163995 | 1 | 655/5150 | 1.15 (1.06, 1.25) | <0.001 |
| Males | 8882/152453 | 1 | 88/1010 | 1.07 (0.87, 1.33) | 0.507 |

^a^Adjusted for age, sex, BMI, smoking status, drinking status, physical activeness, education level, medical history of diabetes, coronary heart disease, chronic kidney disease, heart failure, hypertension, dyslipidemia, anemia, rheumatoid arthritis, the self-reported baseline medication use of steroid and anti-inflammatory drugs, and serum calcium, serum phosphate, and serum vitamin D level.

Supplementary Table 7. Association between the diagnosis of osteoporosis and the risk of incident age-related cataracts in the UK Biobank after excluding cataract cases identified within 2 years of enrolment (n = 337,298)

|  | **No diagnosis of osteoporosis** | | **Diagnosis of Osteoporosis** | | |
| --- | --- | --- | --- | --- | --- |
|  | Case number/ total number | HR (ref) | Case number/ total number | HR^a^ (95% CI) | P-value |
| All individuals | 20601/330800 | 1 | 765/6498 | 1.16 (1.07, 1.24) | <0.001 |
| By sex |  |  |  |  |  |
| Females | 11772/173593 | 1 | 679/5461 | 1.16 (1.07, 1.25) | <0.001 |
| Males | 8829/157207 | 1 | 86/1037 | 1.06 (0.86, 1.31) | 0.591 |

^a^Adjusted for age, sex, BMI, smoking status, drinking status, physical activeness, education level, medical history of diabetes, coronary heart disease, chronic kidney disease, heart failure, hypertension, dyslipidemia, anemia, rheumatoid arthritis, the self-reported baseline medication use of steroid and anti-inflammatory drugs, and serum calcium, and serum phosphate level.

Supplementary Table 9. Results of the multiple mediation analysis in the UK Biobank

|  | HR (95% CI)^a^ | P-value |
| --- | --- | --- |
| Total Effect | 2.30 (1.26, 4.56) | 0.008 |
| Average Direct Effect | 1.52 (0.98, 2.46) | 0.058 |
| Average Causal Mediated Effect^b^ | 1.51 (1.17, 2.02) | 0.002 |
| MEPE | 1.17 (1.02, 1.36) | 0.023 |
| GDF15 | 1.14 (1.03, 1.25) | 0.010 |
| TCN2 | 1.10 (1.00, 1.20) | 0.040 |
| CDCP1 | 1.09 (1.00, 1.21) | 0.044 |
| LRP1 | 1.09 (1.00, 1.19) | 0.064 |
| PLAUR | 1.04 (0.96, 1.14) | 0.298 |
| TFF2 | 0.99 (0.91, 1.09) | 0.900 |
| IL1RL1 | 0.97 (0.93, 1.01) | 0.095 |
| SIGLEC1 | 0.90 (0.82, 0.99) | 0.030 |

^a^Adjusted for age, sex, BMI, smoking status, drinking status and physical activeness.

^b^Total indirect effect through all mediating proteins.

Supplementary Figure 1. Directed Acyclic Graph (DAG) illustrating the multiple mediation model.

*
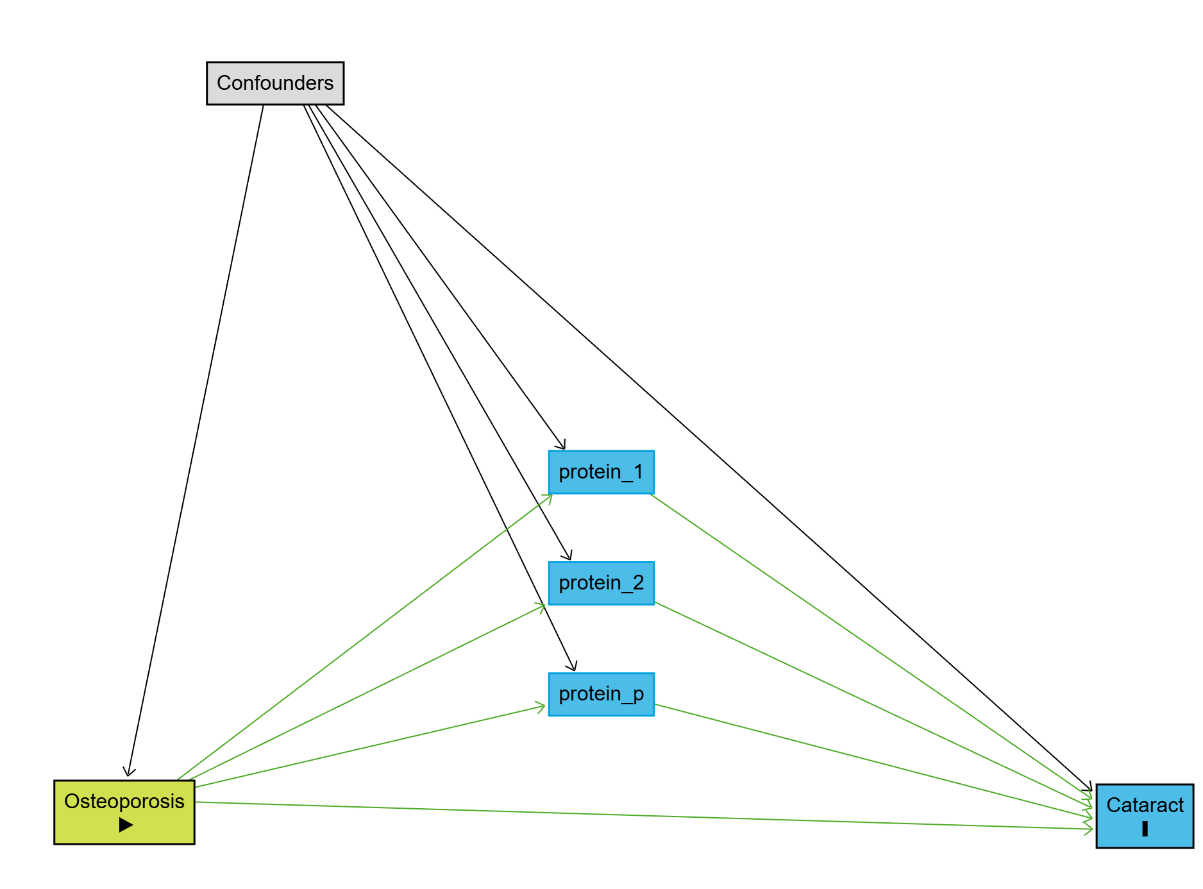
*

This figure depicts a multiple mediation model evaluating whether circulating proteins mediate the association between osteoporosis (exposure) and cataract (outcome). The gray node represents potential confounders (age, sex, BMI, smoking, drinking, physical activity) that may influence the exposure, mediators, and outcome. Green arrows denote hypothesized mediation pathways (osteoporosis → proteins → cataract), while black arrows indicate confounding paths (confounders → exposure, mediators, and outcome). Under the assumptions of mediation analysis, this model aims to estimate the mediating effect of each protein after adjusting for the effect of confounders.
