# Supplementary material for: Sex-specific association between osteoporosis and cataracts: UK Biobank and Hong Kong Osteoporosis Study
Source: Arch Osteoporos. 2026 Feb 24;21(1):44. doi: 10.1007/s11657-026-01674-0 (PMC12932269; doi:10.1007/s11657-026-01674-0)
Supplement: Supplementary file 2 — ESM 2 (PDF 87.1 KB) [file 11657_2026_1674_MOESM2_ESM.pdf]

Supplementary Table 8: Results of the mediation analysis in the UK Biobank.
ACME: Average Causal Mediated Effect
ADE: Average Direct Effect
TE: Total Effect
PM: Percentage Mediated
Mediation model adjusted for age, sex, BMI, smoking status, drinking status and physical activity.
Estimates are differences in survival times in days, as estimated by weibull regression.
Highlighted proteins show evidence of full mediation

Protein	UNIPROT_ID	ACME	ADE	TE	PM	ACME_p	ADE_p	TE_p	PM_p
AREG	P15514	-111.72	-2311.77	-2423.5	4.36	<0.001	0.046	0.036	0.036
ACE2	Q9BYF1	-142.66	-2637.8	-2780.45	4.86	<0.001	0.038	0.036	0.036
ANXA10	Q9UJ72	-130.77	-2650.62	-2781.38	4.59	<0.001	0.02	0.014	0.014
ASGR1	P07306	-130.8	-2728.84	-2859.64	4.38	<0.001	0.02	0.014	0.014
ST6GAL1	P15907	-83.7	-2517.54	-2601.24	3.02	<0.001	0.032	0.026	0.026
BST2	Q10589	-290.27	-2590.39	-2880.67	9.81	<0.001	0.026	0.014	0.014
REN	Q693B1	-125.03	-2501.21	-2626.24	4.54	<0.001	0.028	0.022	0.022
CCL15	Q16663	-115.9	-2502.03	-2617.94	4.19	<0.001	0.03	0.026	0.026
CCL20	P78556	-175.53	-2445.25	-2620.78	6.54	<0.001	0.036	0.018	0.018
CXCL17	Q6UXB2	-124.67	-2556.79	-2681.45	4.56	<0.001	0.022	0.012	0.012
CPM	P14384	-130.95	-2645.07	-2776.02	4.61	<0.001	0.012	0.012	0.012
CHGA	P10645	-336.25	-2871.13	-3207.39	10.38	<0.001	0.012	0.002	0.002
CDCP1	Q9H5V8	-230.82	-2655.4	-2886.22	7.8	<0.001	0.018	0.008	0.008
FABP4	P15090	-134.45	-2529.63	-2664.08	4.78	<0.001	0.032	0.022	0.022
LGALS4	P56470	-234.24	-2347.81	-2582.05	8.97	<0.001	0.038	0.02	0.02
GAST	P01350	-279.54	-3182.93	-3462.47	8.04	<0.001	0.016	0.006	0.006
GGT1	P19440	-117.51	-2788.26	-2905.77	3.89	<0.001	0.022	0.012	0.012
PLA2G10	O15496	-101.4	-2742.35	-2843.75	3.41	<0.001	0.02	0.014	0.014
GDF15	Q99988	-466.29	-2201.13	-2667.42	17.28	<0.001	0.056	0.016	0.016
HAVCR1	Q96D42	-224.66	-2715.28	-2939.94	7.4	<0.001	0.026	0.016	0.016
HAVCR2	Q8TDQ0	-161.98	-2786.47	-2948.45	5.36	<0.001	0.02	0.016	0.016
IL1RL1	Q01638	-181.66	-2348.08	-2529.74	6.91	<0.001	0.06	0.032	0.032
IL1R1	P14778	-123.4	-2866.79	-2990.19	4.03	<0.001	0.008	0.006	0.006
IL10	P22301	-121.14	-2278.24	-2399.38	4.72	<0.001	0.066	0.048	0.048
IL15	P40933	-334.48	-2179.6	-2514.08	12.82	<0.001	0.06	0.03	0.03
IL18R1	Q13478	-145.85	-2446.61	-2592.46	5.57	<0.001	0.03	0.02	0.02
IL6	P05231	-110.47	-2402.38	-2512.85	4.25	<0.001	0.048	0.04	0.04
SPINT1	O43278	-173.97	-2913.58	-3087.55	5.55	<0.001	0.004	0.002	0.002
REG1A	P05451	-121.01	-2750.53	-2871.54	4.12	<0.001	0.01	0.006	0.006
MMP12	P39900	-93.22	-2860.86	-2954.08	3.11	<0.001	0.016	0.016	0.016
MSR1	P21757	-161.29	-2767.63	-2928.92	5.41	<0.001	0.012	0.008	0.008
MMP7	P09237	-148.48	-2527.4	-2675.88	5.33	<0.001	0.032	0.022	0.022
MEPE	Q9NQ76	-315.45	-2102.49	-2417.93	12.35	<0.001	0.072	0.04	0.04
NFASC	O94856	-106.09	-2546.31	-2652.4	3.84	<0.001	0.028	0.022	0.022

OCLN	Q16625	-212.11	-3230.9	-3443.01	6.09	<0.001	0.01	0.002	0.002
PLXNB2	O15031	-111.79	-2304.09	-2415.88	4.47	<0.001	0.038	0.026	0.026
PIGR	P01833	-264.66	-2890.97	-3155.64	8.28	<0.001	0.014	0.006	0.006
ADM	P35318	-151.24	-2675.77	-2827.01	5.32	<0.001	0.014	0.006	0.006
LRP1	Q07954	-204.63	-2218.98	-2423.62	8.1	<0.001	0.072	0.044	0.044
DPY30	Q9C005	-110.82	-2857.58	-2968.4	3.64	<0.001	0.014	0.004	0.004
REG3A	Q06141	-189.51	-2424.29	-2613.8	6.96	<0.001	0.04	0.024	0.024
REG4	Q9BYZ8	-175.94	-2365.89	-2541.82	6.74	<0.001	0.036	0.028	0.028
REN	P00797	-125.03	-2501.21	-2626.24	4.54	<0.001	0.028	0.022	0.022
RNASET2	O00584	-132.93	-2384.36	-2517.3	5.04	<0.001	0.04	0.026	0.026
RRM2	P31350	-108.54	-2768.14	-2876.69	3.67	<0.001	0.018	0.014	0.014
SIGLEC1	Q9BZZ2	-103.56	-2425.93	-2529.48	3.91	<0.001	0.03	0.022	0.022
CD80	P33681	-143.91	-2988.24	-3132.15	4.5	<0.001	0.016	0.012	0.012
TCN2	P20062	-161.36	-2443.83	-2605.18	5.91	<0.001	0.038	0.024	0.024
TFF2	Q03403	-200.37	-2311.09	-2511.46	7.86	<0.001	0.044	0.032	0.032
TREM2	Q9NZC2	-89.58	-2465.75	-2555.33	3.28	<0.001	0.032	0.024	0.024
TNFRSF10A	O00220	-172.03	-2692.15	-2864.18	5.91	<0.001	0.018	0.018	0.018
TNFRSF10[illegible]	O14763	-94.19	-2720.47	-2814.65	3.25	<0.001	0.012	0.01	0.01
EDA2R	Q9HAV5	-137.09	-2612.55	-2749.64	4.83	<0.001	0.03	0.018	0.018
PLAUR	Q03405	-213.74	-2309.05	-2522.79	8.19	<0.001	0.048	0.04	0.04
VSIG2	Q96IQ7	-286.84	-3236.95	-3523.79	7.92	<0.001	0.01	0.006	0.006
VSIG4	Q9Y279	-146.16	-2779.43	-2925.59	4.85	<0.001	0.014	0.008	0.008
WFDC2	Q14508	-193.46	-2687.93	-2881.39	6.59	<0.001	0.016	0.016	0.016
WFIKKN1	Q96NZ8	-115.41	-2743.39	-2858.81	4.02	<0.001	0.018	0.014	0.014
